# Downregulation of miR-193a-3p is involved in the pathogenesis of hepatocellular carcinoma by targeting CCND1

**DOI:** 10.7717/peerj.8409

**Published:** 2020-02-10

**Authors:** Shi-shuo Wang, Zhi-guang Huang, Hua-yu Wu, Rong-quan He, Li-hua Yang, Zhen-bo Feng, Yi-wu Dang, Hui-ping Lu, Ye-ying Fang, Gang Chen

**Affiliations:** 1 Department of Pathology, First Affiliated Hospital of Guangxi Medical University, Nanning, Guangxi, China; 2 Department of Cell Biology & Genetics, Guangxi Medical University, Nanning, Guangxi, China; 3 Department of Medical Oncology, First Affiliated Hospital of Guangxi Medical University, Nanning, Guangxi, China; 4 Department of Radiation Oncology, Radiation Oncology Clinical Medical Research Center of Guangxi, First Affiliated Hospital of Guangxi Medical University, Nanning, Guangxi, China

**Keywords:** Mir-193a-3p, Hepatocellular Carcinoma, Cyclin D1, Cell proliferation, Apoptosis, Bioinformatics

## Abstract

**Background:**

Hepatocellular carcinoma (HCC) is the second-highest cause of malignancy-related death worldwide, and many physiological and pathological processes, including cancer, are regulated by microRNAs (miRNAs). miR-193a-3p is an anti-oncogene that plays an important part in health and disease biology by interacting with specific targets and signals.

**Methods:**

In vitro assays were performed to explore the influences of miR-193a-3p on the propagation and apoptosis of HCC cells. The sequencing data for HCC were obtained from The Cancer Genome Atlas (TCGA), and the expression levels of miR-193a-3p in HCC and non-HCC tissues were calculated. The differential expression of miR-193a-3p in HCC was presented as standardized mean difference (SMD) with 95% confidence intervals (CIs) in Stata SE. The impact of miR-193a-3p on the prognoses of HCC patients was determined by survival analysis. The potential targets of miR-193a-3p were then predicted using miRWalk 2.0 and subjected to enrichment analyses, including Gene Ontology (GO) annotation, Kyoto Encyclopedia of Genes and Genomes (KEGG) pathway analysis, and Protein-Protein Interaction (PPI) network analysis. The interaction between miR-193a-3p and one predicted target, Cyclin D1 (CCND1), was verified by dual luciferase reporter assays and Pearson correlation analysis.

**Results:**

MiR-193a-3p inhibited the propagation and facilitated the apoptosis of HCC cells *in vitro*. The pooled SMD indicated that miR-193a-3p had a low level of expression in HCC (SMD: −0.88, 95% CI [−2.36 −0.59]). Also, HCC patients with a higher level of miR-193a-3p expression tended to have a favorable overall survival (OS: HR = 0.7, 95% CI [0.43–1.13], *P* = 0.14). For the KEGG pathway analysis, the most related pathway was “proteoglycans in cancer”, while the most enriched GO term was “protein binding”. The dual luciferase reporter assays demonstrated the direct interaction between miR-193a-3p and CCND1, and the Pearson correlation analysis suggested that miR-193a-3p was negatively correlated with CCND1 in HCC tissues (*R* =  − 0.154, *P* = 0.002).

**Conclusion:**

miR-193a-3p could suppress proliferation and promote apoptosis by targeting CCND1 in HCC cells. Further, miR-193a-3p can be used as a promising biomarker for the diagnosis and treatment of HCC in the future.

## Introduction

Hepatocellular carcinoma (HCC) is the most prevalent type of primary liver cancer, and it accounts for approximately 70 to 90% of liver cancer cases. In terms of morbidity, it ranks fifth among males, ninth among females, and second among factors for cancer death worldwide (Schoenberg et al., 2018). Hepatocellular carcinoma occurs in the environment of chronic hepatitis and is highly associated with hepatitis viral infection (hepatitis B or C) or toxin exposure (e.g., alcohol and aflatoxins). For patients with chronic hepatitis B or C, even effective antiviral therapy cannot completely eliminate the risk of HCC occurrence or relapse after effective treatment. However, the molecular mechanism of HCC remains poorly understood, and it is necessary to explore the effective biomarkers for HCC. MicroRNAs (miRNAs) control a variety of physiological and pathological processes, including cancer. MiRNAs have been shown to be directly involved in the cell proliferation, apoptosis, and metastasis of HCC by targeting many key protein-coding genes (Huang & He, 2011). The aberrant expression of miRNAs and their corresponding target genes may be essential in the initiation and development of hepatic carcinoma.

MiRNAs are internal non-coding small RNAs regulating protein-coding genes in a sequence-specific manner (Shi, Han & Spivack, 2013), and they adjust the expression of numerous genes by attaching to specific sequences in the 3′-untranslated region (UTR). This results in mRNA degradation or the suppression of mRNA translation (Moura, Borsheim & Carvalho, 2014). Multiple studies have shown that miRNAs participate in the invasion and metastasis of HCC (Di Leva, Briskin & Croce, 2012; Sun et al., 2013). Some miRNAs could also function as biomarkers for early diagnosis, stratification, and response evaluation in HCC (Braconi & Patel, 2008). Chen et al. (2018) demonstrated that miR-590-5p inhibited HCC chemoresistance by targeting yes-associated protein 1 (YAP1). MiR-133b promotes cell proliferation and metastasis in HCC by regulating splicing factor 3b subunit 4 (SF3B4) (Liu et al., 2018b). MiR-3650 suppresses HCC migration and epithelial-mesenchymal transition (EMT) by directly targeting neurofascin (Wu et al., 2019). Targeting miR-494-3p, miR-126-3p, or miR-342-3p could inhibit HCC invasion and metastasis (Liu et al., 2018a; Lou et al., 2018). Gu et al. (2019) concluded that the miR-144/CCNB1 (Cyclin B1) axis was important and that the inhibition of miR-144 could improve the outcomes of human HCC. MiR-140-3p increases the sensitivity of HCC cells to sorafenib (Li et al., 2018). A previous study showed that miR-193a-3p expression was regulated by DNA promoter hypermethylation and miR-193a-3p facilitated HCC resistance to 5-fluorouracil (5-FU) via repressing serine/arginine-rich splicing factor 2 (SRSF2) (Ma et al., 2012). Our team found that miR-193a-3p was downregulated in HCC by means of in-house quantitative reverse transcription polymerase chain reactions (qRT-PCR) in 2015, and it could also serve as a prognostic indicator (Liu et al., 2015). However, in terms of expression, there are no research papers on HCC concerning the correlation between miR-193a-3p and its target genes. Therefore, we forecasted the target genes of miR-193a-3p in the present study.

Previous studies have demonstrated that miR-193a-3p inhibits the expression of some carcinogenic factors, such as CDK 6, c-kit, E2F6, and E-cadherin, which could enhance tumor invasion and angiogenesis (Gao et al., 2011; Iliopoulos, Rotem & Struhl, 2011; Liu et al., 2015; Ma et al., 2012). Cyclin, a group of regulatory subunits of the holoenzyme, are able to regulate cellular processes through the cell cycle (Liu et al., 2017). As a proto-oncogene (Karimkhanloo et al., 2017), CCND1 can promote DNA synthesis, cell proliferation, cell colonization, and hepatoma formation (Wu, Lan & Liu, 2019). Several studies have confirmed that miR-193a-3p inhibits the growth of gastric and prostate cancer cells by targeting CCND1 (Chou et al., 2018; Liu et al., 2017), but its role in HCC remains unclear. Therefore, we studied the relationship between miR-193a-3p and its target genes in HCC, as well as the potential mechanism involved.

We performed in vitro assays to explore the effect of miR-193a-3p on the propagation and apoptosis of HCC cells. We then used the sequencing data of HCC to determine the expression of miR-193a-3p and CCND1. The impact of miR-193a-3p on the prognoses of patients with HCC was evaluated by survival analysis. Finally, it was determined that miR-193a-3p suppressed the proliferation and promoted the apoptosis of HCC cells, and miR-193a-3p has a negative correlation with CCND1 in HCC. For the first time, the interconnection between their expression levels in HCC was mined in depth, and the underlying molecular mechanism of miR-193a-3p in HCC was studied.

## Materials & Methods

### Re-expression and suppression of miR-193a-3p in HCC cells

The HCC cell line Hep3B was cultured at 37 °C in Dulbecco’s Modified Eagle Medium (Thermo Fisher Scientific, Waltham, MA, USA) supplemented with 10% fetal bovine serum, 100 units/ml penicillin, and 100 ug/ml streptomycin in a humid incubator with 5% CO2. In accordance with the manufacturer’s procedure, we used CombiMag Magnetofection (OZ Biosciences, Marseille Cedex 9, France) to transfect Hep3B cells with the miR-193a-3p inhibitor (sequence: 5′-ACUGGGACUUUGUAGGCCAGUU-3′), the miRNA inhibitor’s negative control (sequence: 5′-ACGUGACACGUUCGGAGAATT-3′), the simulated miR-193a-3p (sequence: 5′-AACUGGCCUACAAAGUCCCAGU-3′), and the negative control for the miRNA simulation (sequence: 5′-UUCUCCGAACGUGUCACGUTT-3′) (GenePharma, Shanghai, China) at an equal concentration of 20 µM (20 pmol/µl). Hep3B cells were seeded into a 96-well plate at a density of 3 × 10^4^/well. After being transfected with miRNA simulations or miRNA inhibitors, cells were cultivated for ten days. Among them, zero-day samples and intermediate samples at the fifth day were gathered and analyzed via diverse experiments. A cell proliferation assay was performed using a Promega MTS kit (cat. no. G3580; Promega Corporation, Beijing, China). For the apoptosis assay, cells were double-stained with Hoechst 33,342/propidium iodide (PI) kit (Sigma-Aldrich Co., St Louis, MO, USA) according to the manufacturer’s instructions. The viable and apoptotic cells were observed and counted under a fluorescence microscope (100×, ZEISS Axiovert 25, Zaventem, Belgium).

### qRT-PCR

The expression of miR-193a-3p was then confirmed via qRT-PCR. Total RNA Kit I (Promega, Beijing, China) was used to extract total RNA from the above-mentioned cells. The tailing reaction miRNA First Strand cDNA Synthesis (Sangon Biotech, Shanghai, China) was used to inversely transcribe cDNA. The primer sequence for miR-193a-3p was 5′- AACUGGCCUACAAAGUCCCAGU-3′. The primer sequence for U6 (internal control) was 5′-ACACTCCAGCTGGGAACTGGCCTACAAAGTCC-3′. The PCR was performed using an SYBR Green MicroRNAs qPCR Kit (SYBR Green Method) (Sangon Biotech) on an ABI Prism 7500 (Applied Biosystems, Foster City, CA, USA). The expression of miR-193a-3p in each group was initially calculated via the 2$\hat {}$^(−ΔΔCq)^ method and normalized to the mock group (cells that were transfected solely with CombiMag Magnetofection). Subsequently, the change in miR-193a-3p level upon transfection was calculated with the formula (2$\hat {}$^ΔΔCq^-1) (Chen et al., 2012).

### Differential expression of miR-193a-3p in HCC in miRNA-sequencing data

We downloaded sequencing data for miR-193a-3p’s expression profile in HCC from The Cancer Genome Atlas (TCGA) on May 1, 2019, including 369 HCC tissues and 49 non-HCC tissues. We also used the in-house qRT-PCR results of our team’s work in 2015, which contained 95 HCC tissues and 95 non-HCC tissues. The Medical Ethics Committee of First Affliated Hospital of Guangxi Medical University approved this study (approval no. 2015 KY-E-041). Each participant signed an informed consent. We computed the number, mean, and standard deviation of miR-193a-3p expression levels in cancer and non-cancer groups. Next, we used StataSE (StataCorp, College Station, TX, USA), SPSS 25.0 (IBM, Armonk, New York) and GraphPad Prism 8 software (GraphPad Software Inc., San Diego, CA, US) for the statistical analyses. By comparing the expression in HCC and non-HCC tissues with standardized mean difference (SMD) and a violin plot, the differential expression of miR-193a-3p in HCC and non-HCC tissues was confirmed, and its diagnostic significance was evaluated. We also retrieved the expression data for CCND1 in HCC from 371 HCC and 50 non-HCC samples in the form of RNA sequencing data. The association between miR-193a-3p and CCND1 was analyzed using these downloaded data in SPSS 25.0, and *P* < 0.05 was defined as statistically significant.

### Relationship between miR-193a-3p and progression of HCC

We retrieved the Kaplan–Meier plots from the Kaplan–Meier Plotter website (http://kmplot.com/) to analyze the survival discrepancies between patients with high and low expression levels of miR-193a-3p and further evaluate its prognostic value in HCC.

### Enrichment analyses and a protein-protein interaction (PPI) network construction

To further explore the potential molecular mechanism of miR-193a-3p in HCC, we conducted a bioinformatics analysis. First, the potential targets of miR-193a-3p were predicted using miRWalk 2.0 (http://zmf.umm.uni-heidelberg.de/mirwalk2), a comprehensive database comprised of twelve predictive online tools: microT-CDSmiRWalk, MicroT4microT4, miRanda, miRBridge, miRDB, miRMap, miRNAMap, PICTAR2, PITA, RNA22, RNAhybrid, and TargetScan. Genes that appeared in more than five platforms were subjected to subsequent analysis. The Kyoto Encyclopedia Genes and Genomes (KEGG) pathway terms and Gene Ontology (GO) biological processes, cellular components, and molecular functions were analyzed using the Database for Annotation, Visualization, and Integrated Discovery (DAVID 6.7) (https://david.ncifcrf.gov/). We defined the significant GO and KEGG pathways with the criterion of *P* < 0.05.

To reveal the link between the target genes, we constructed a PPI network using STRING software v10.0 (https://string-db.org/). Based on the number of nodes and edges, the genes most related to miR-193a-3p were identified. Hub genes were identified by the numerical digits of the degrees of every node and edge. *P* < 0.05 was considered statistically significant. Among the hub genes, we selected CCND1 as the target gene for miR-193a-3p in this study, and the relationship between them was explored in depth.

### Dual-Luciferase reporter experiments

We used the Dual-Luciferase® Reporter Assay System (Promega, WI, USA) to identify the activity of luciferase and the target gene’s 3′UTR plasmids (CCND1 3′UTR + miR-193a-3p) or no-load plasmids (CCND1 3′UTR-NC + miR-193a-3p) in transfecting HEK293T cells. To make the experimental results more reliable, we set up a positive reference miRNA group (has-miR-146b vector plasmid) and a positive reference miRNA NC (negative control) group (TRAF6 3′UTR plasmids).

### Statistical analysis

We performed an independent samples *t*-test and a Pearson correlation analysis (two-tailed) using SPSS 25.0 to analyze the data we extracted from the miRNA-sequencing data and the previous in-house qRT-PCR data. We considered *P* < 0.05 to indicate statistical significance. We also used StataSE to perform the meta-analysis, and the combined SMD with 95% confidence intervals (CIs) were computed to estimate the continuous outcomes. Violin plots and bar charts were generated using GraphPad Prism 8. One-way analysis of variance (ANOVA) and the post-hoc Bonferroni test were applied in the *in vitro* experiments to draw comparisons between groups in GraphPad Prism8. We performed GO and KEGG pathway analyses through DAVID and the R package, using ‘GOplot’ and ‘ggplot2’ to visualize the results. In the meantime, based on bioinformatics predictions, two binding sites of miR-193a-3p on CCND1 and the negative correlation between the two genes in various cancers were found on starBase v3.0 (http://starbase.sysu.edu.cn/index.php).

## Results

### miR-193a-3p inhibited cell propagation and promoted apoptosis *in vitro*

**Figure 1:**
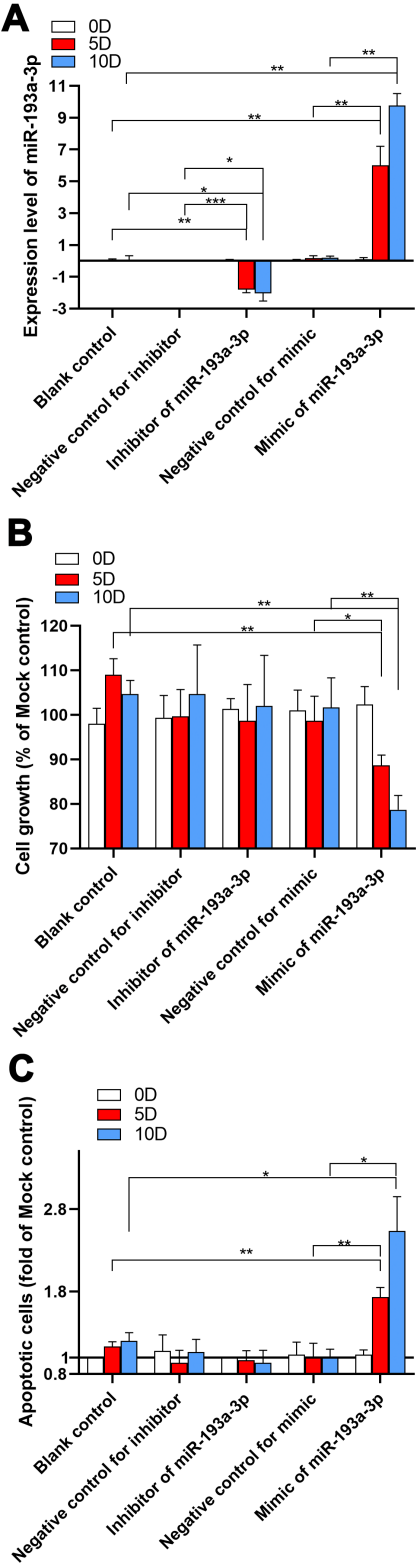
Results of in vitro experiments of miR-193a-3p in HCC. (A) Expression level of miR-193a-3p in HCC cell line Hep3B. Hep3B cells were treated with inhibitor or mimic of miR-193-3p or negative control and the alteration of miR-193-3p expression was confirmed by qRT-PCR. (B) Effect of miR-193a-3p on proliferation of Hep3B cells as determined by Hoechst 33342/propidium iodide (PI) dual-luciferase chromatin staining. (C) Effect of miR-193a-3p on apoptosis in Hep3B cells as determined by Hoechst 33342/propidium iodide (PI) dual-luciferase chromatin staining. Hep3B cells were cultured with miR-193a-3p inhibitor, mimic or different controls for 0, 5, and 10 days, and using the CellTiter-Blue cell viability assay. *P < 0.05, **P < 0.01, ***P < 0.0001, compared to blank control or negative control at the same day.

Compared with the blank control and the negative simulated control group, the miR-193a-3p level in Hep3B cells that treated with miR-193a-3p inhibitor was significantly decreased at five days after transfection (*P* < 0.001). In contrast, the transfection of the miR-193a-3p simulation led to a sharp increase in miR-193a-3p level within five and ten days (*P* < 0.01) (Fig. 1A). To determine the effect of miR-193a-3p on cell proliferation, viable (Hoechst 33342 positive/PI negative) cells were counted after transfection under a microscope. We observed that upon miR-193a-3p’s simulation, the amount of cell proliferation significantly decreased at five and ten days (*P* < 0.01) (Fig. 1B). Apoptotic assays showed that upon the upregulation of miR-193a-3p, the apoptotic cells increased 1.4 folds. However, under inhibitor action, the quantity of apoptotic cells decreased slightly in the Hep3B cells, though this change was not significant (Fig. 1C).

### miRNA expression level and its clinical significance in HCC

**Figure 2:**
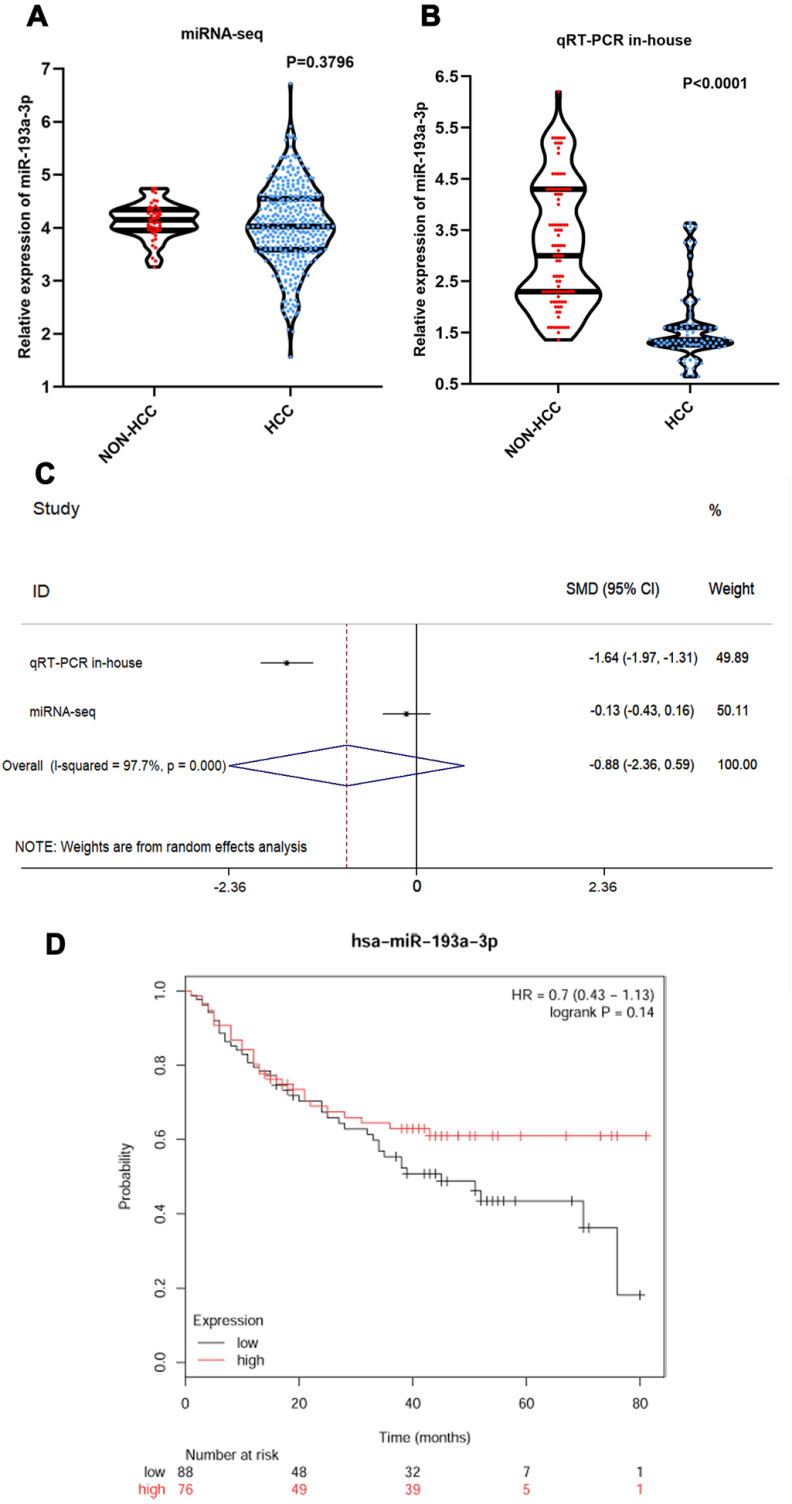
The expression of miR-193-3p and its clinical significance in HCC. (A) Violin plot of miR-193a-3p expression in HCC based on TCGA database. (B) Violin plot of miR-193a-3p expression in HCC based on in-house qRT-PCR. (C) Forest plot of miR-193a-3p expression in HCC (random-effects model). SMD < 0 indicates that miR-193-3p was downregulated in HCC tissues as compared with non-tumor tissues. (D) Overall survival (OS) analysis in Kaplan-Meier Plotter online website.

Four hundred and sixty-four HCC and 144 non-tumor tissue samples were collected from miRNA-sequencing data (online) and the in-house qRT-PCR. We used violin plots to visualized the differential expression of miR-193a-3p. Based on the data for miRNA sequencing, no statistical difference in miR-193a-3p level was observed between HCC and non-tumor tissues (*P* = 0.3796) (Fig. 2A). In contrast, miR-193-3p was downregulated in HCC tissues based on the results of in-house qRT-PCR (*P* < 0.0001) (Fig. 2B). The pooled result from a random-effects model (*I*^2^ = 97.7%; *P* = 0.000) indicated that miR-193a-3p was expressed at a low level in HCC (SMD: -0.88; 95% CI: -2.36–0.59) (Fig. 2C). The Kaplan–Meier plot indicated that HCC patients with a higher level of miR-193a-3p expression tended to have a more favorable overall survival rate than those with a low level of expression, though the difference was not significant (OS: HR=0.7, 95% CI [0.43–1.13], *P* = 0.14) (Fig. 2D).

### Enrichment analyses of candidate target genes and PPI network construction

**Figure 3:**
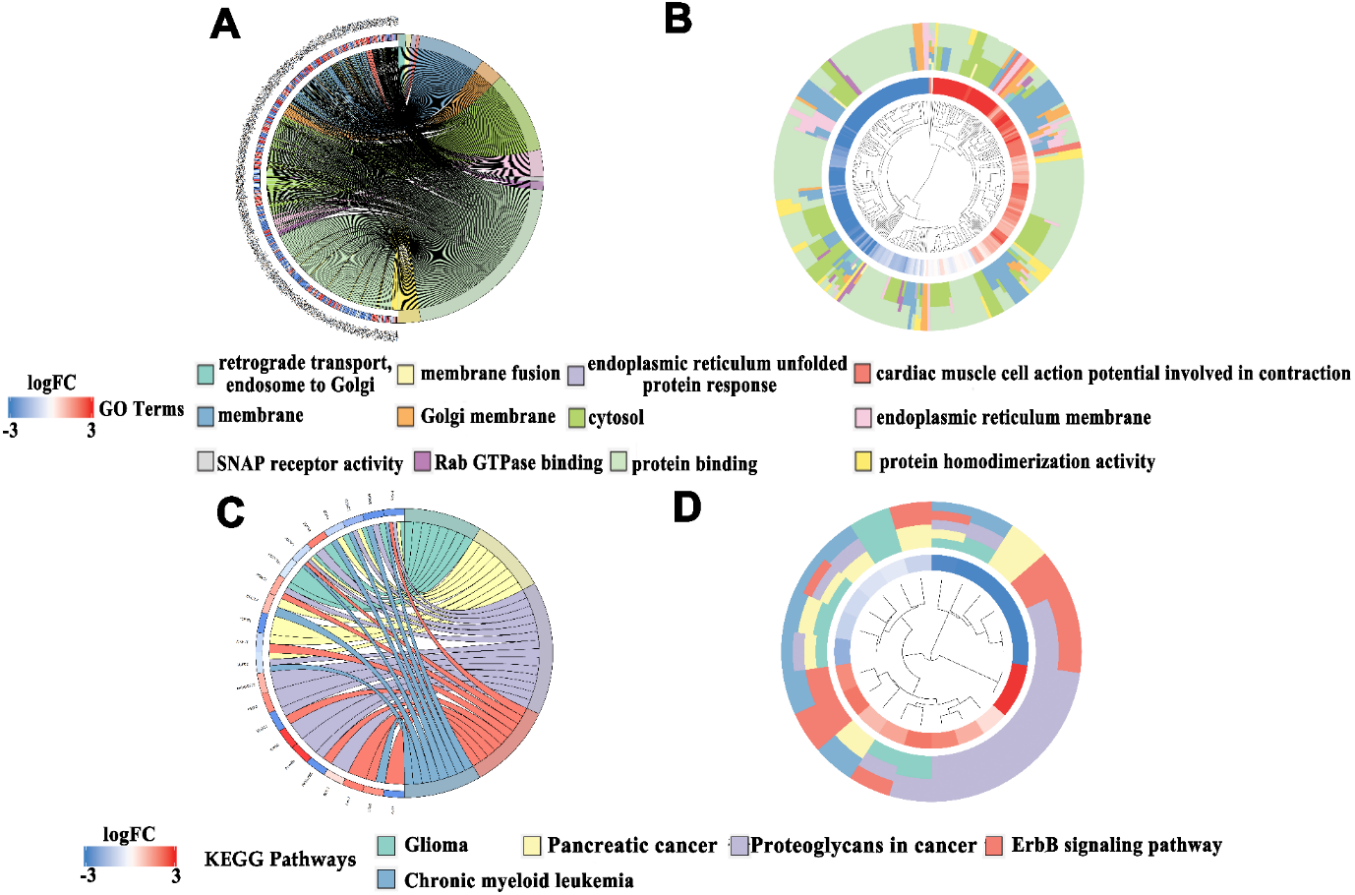
Enrichment analysis of miR-193a-3p related genes in the KEGG and GO pathways. (A) The related genes in a Chord plot are linked to their enriched GO annotations via ribbons. (B) A cluster plot shows a circular dendrogram of the clustering analysis of expression profiles. The inner ring shows the color-coded logFC, while the outer ring shows the GO annotations. (C) Chord plot of KEGG pathways. (D) Cluster plot of KEGG pathways. Red codes next to the selected genes indicate upregulation, and blue codes indicate downregulation.

**Figure 4:**
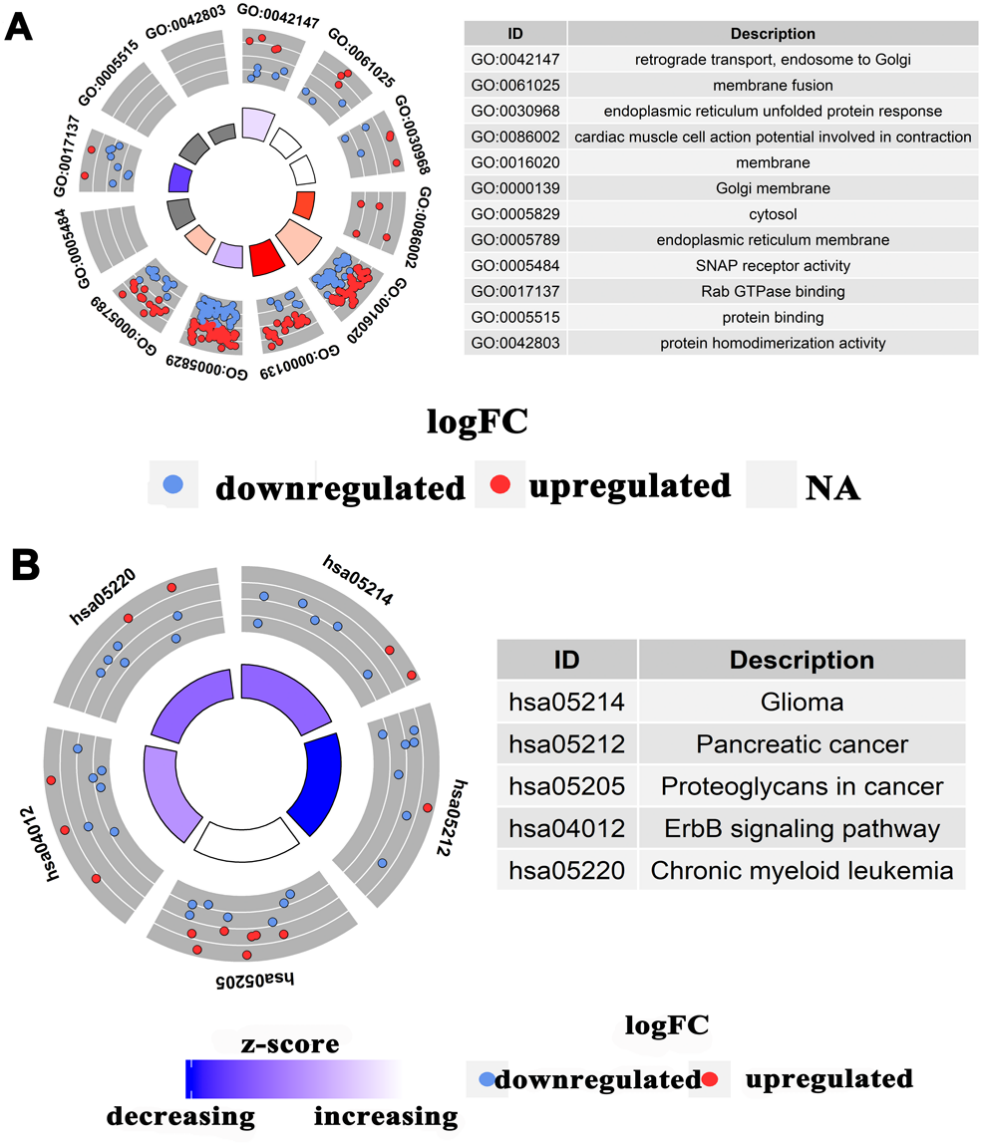
Concentric circle diagram of the GO analysis and KEGG pathways. (A) Concentric circle diagram of the GO analysis. The nodes in the concentric circle represent the co-expression genes clustered in the GO annotations. (B) Concentric circle graph of KEGG pathways. The larger and darker areas in the inner circle are more abundant.

**Figure 5:**
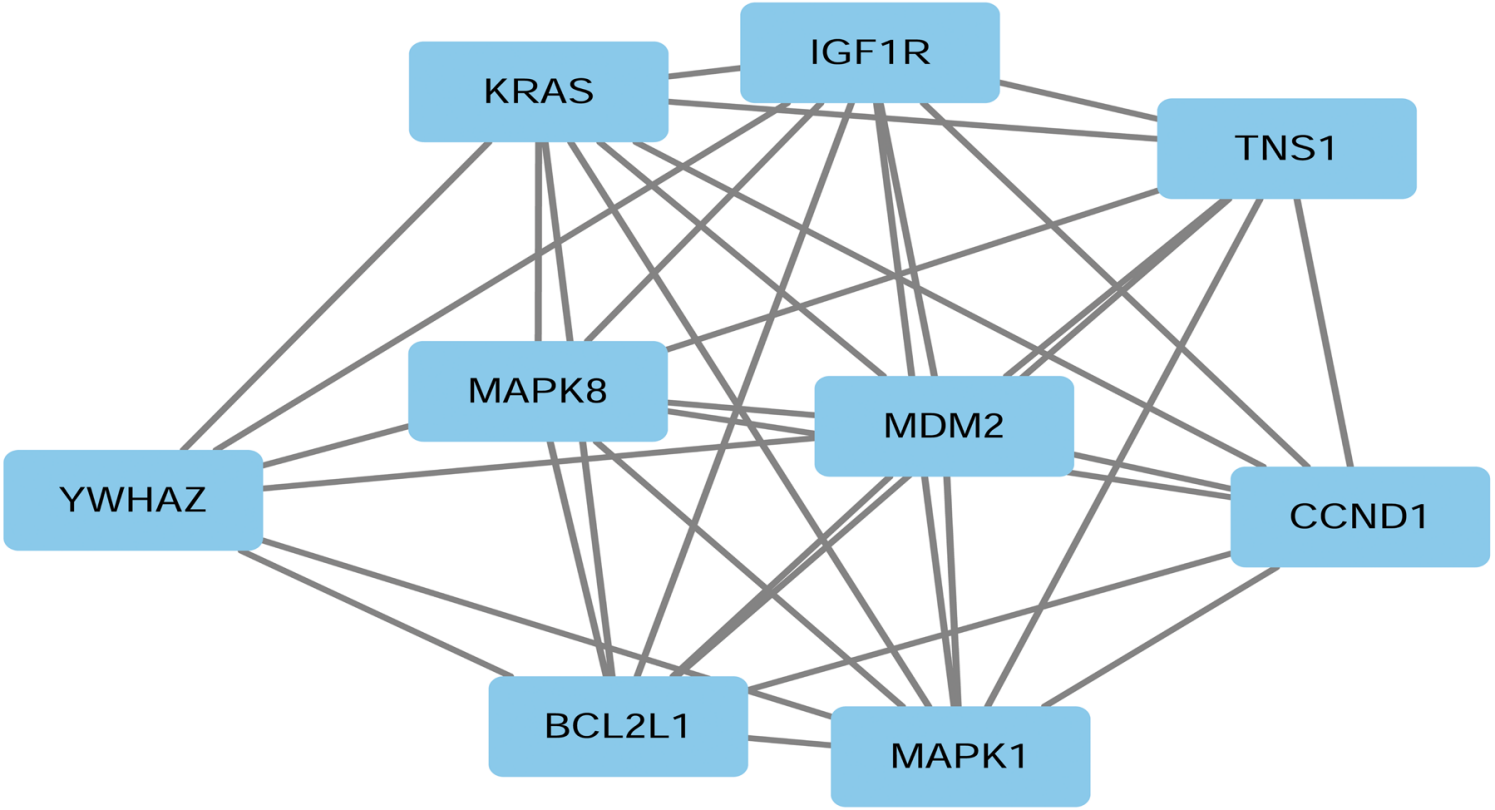
PPI network of nine representative target genes of miR-193a-3p in HCC. The proteins in the blue rectangular boxes are hub genes, while the gray continuous lines represent interactions between individual proteins.

We conducted GO and KEGG pathway analyses to study the functional connections between the genes related to miR-193a-3p. Table 1 shows the top four significant gene annotations in GO and the top five pathways in KEGG regarding the potential target of miR-193a-3p. As can be seen in Figs. 3 and 4, the KEGG pathway analysis showed that “Proteoglycans in cancer” is the most closely related pathway, while the “ErbB signaling pathway” is the second most closely related pathway. Among all the miR-193a-3p-related genes, the most enriched GO term is “protein binding”. The PPI network in Fig. 5 showed the nine genes most related to miR-193-3p, including KRAS, IGF1R, TNS1, YWHAZ, MAPK8, MDM2, BCL2L1, MAPK1, and CCND1. Based on the enrichment analyses and the constructed PPI network, CCND1 was selected as the target of miR-193a-3p for the subsequent research.

### Dual-luciferase reporter assays verified the interaction between miR-193a-3p and CCND1

**Figure 6:**
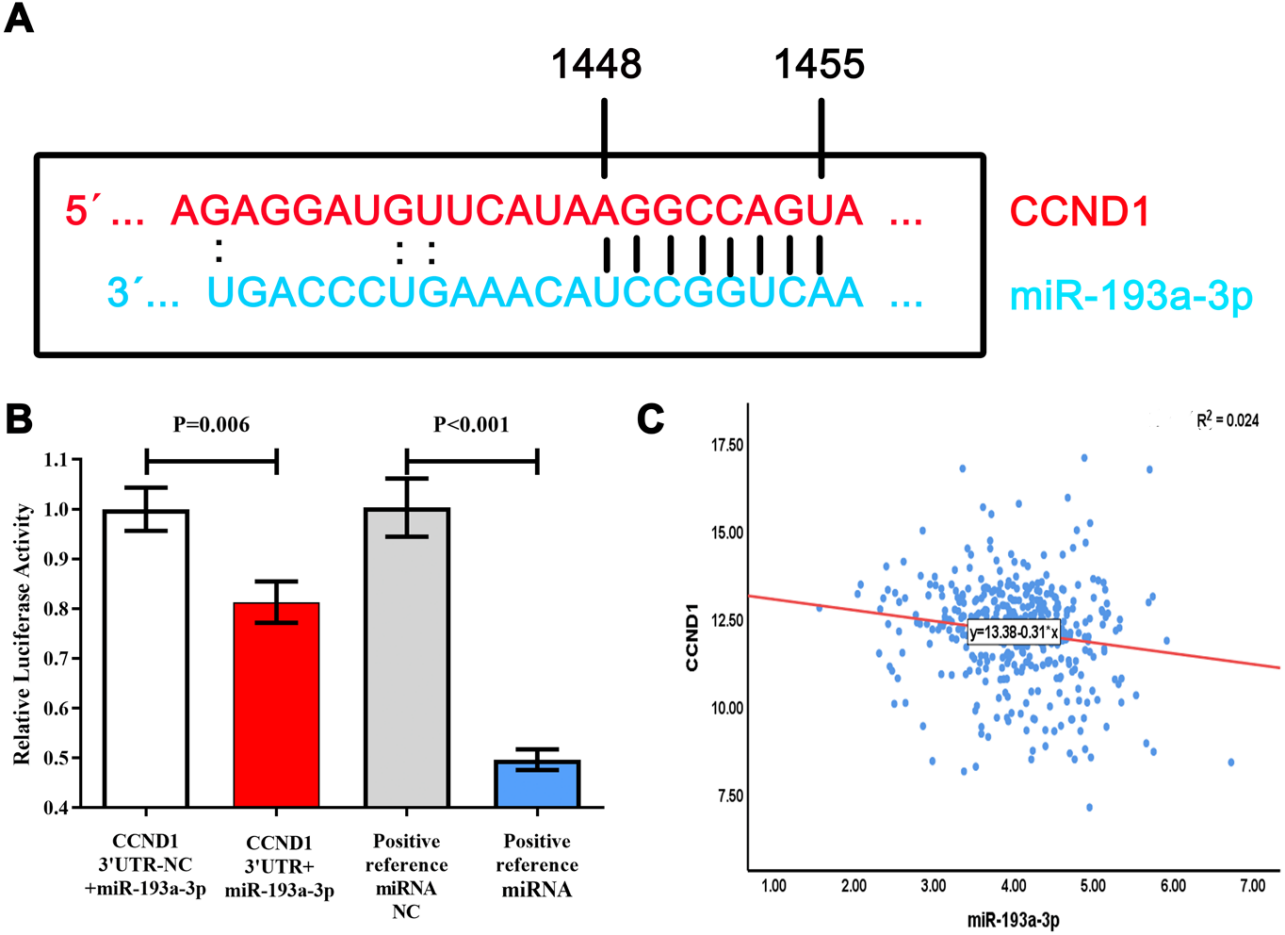
Relationship between miR-193a-3p and CCND1. (A) Complementary base sequences of miR-193a-3p and CCND1. (B) The relative luciferase activity of CCND1 3′UTR+miR-193a-3p decreased as compared with CCND1 3′UTR-NC + miR-193a-3p cells. The relative luciferase activity of the positive control miRNA group was lower than that of the positive control miRNA NC group. (C) Negative correlation between miR-193a-3p and CCND1 in TCGA database-Pearson correlation analysis (double-tailed). Positive reference miRNA group: hsa-miR-146b vector plasmid; positive reference miRNA NC (negative control) group: TRAF6 3′UTR plasmids.

Through a search on miRwalk 2.0, we found the base-complementary pairing between miR-193a-3p and CCND1 (Fig. 6A). Subsequently, double luciferase reporter assays verified the direct interaction between miR-193a-3p and CCND1. The relative luciferase activity of the CCND1 3′UTR+miR-193a-3p group decreased as compared with CCND1 3′UTR-NC +miR-193a-3p cells (*P* = 0.006) (Fig. 6B). The relative luciferase activity of the positive-control miRNA group was lower than that of the positive-control miRNA NC group (*P* < 0.001) (Fig. 6B).

### Expression of miR-193a-3p was negatively correlated with that of CCND1

**Figure 7:**
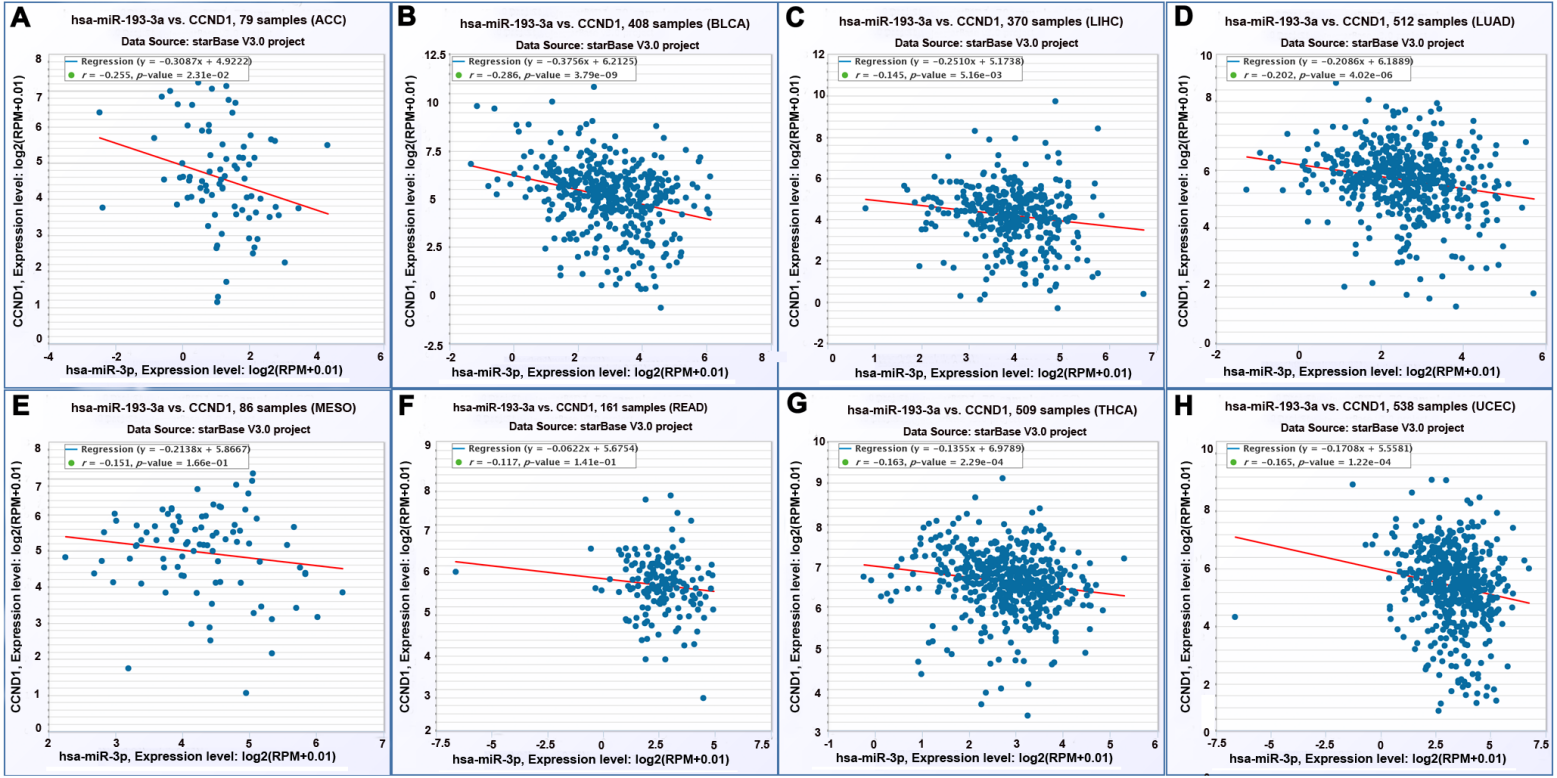
Correlation between miR-193a-3p and CCND1 on the basis of starBase v3.0 pan-cancer analysis project (binding site: chr11: 69467239-69467244[+]). (A) Adrenocortical carcinoma. (B) Bladder urothelial carcinoma. (C) Liver hepatocellular carcinoma. (D) Lung adenocarcinoma. (E) Mesothelioma. (F) Rectum adenocarcinoma. (G) Thyroid carcinoma. (H) Uterine corpus endometrial carcinoma.

**Figure 8:**
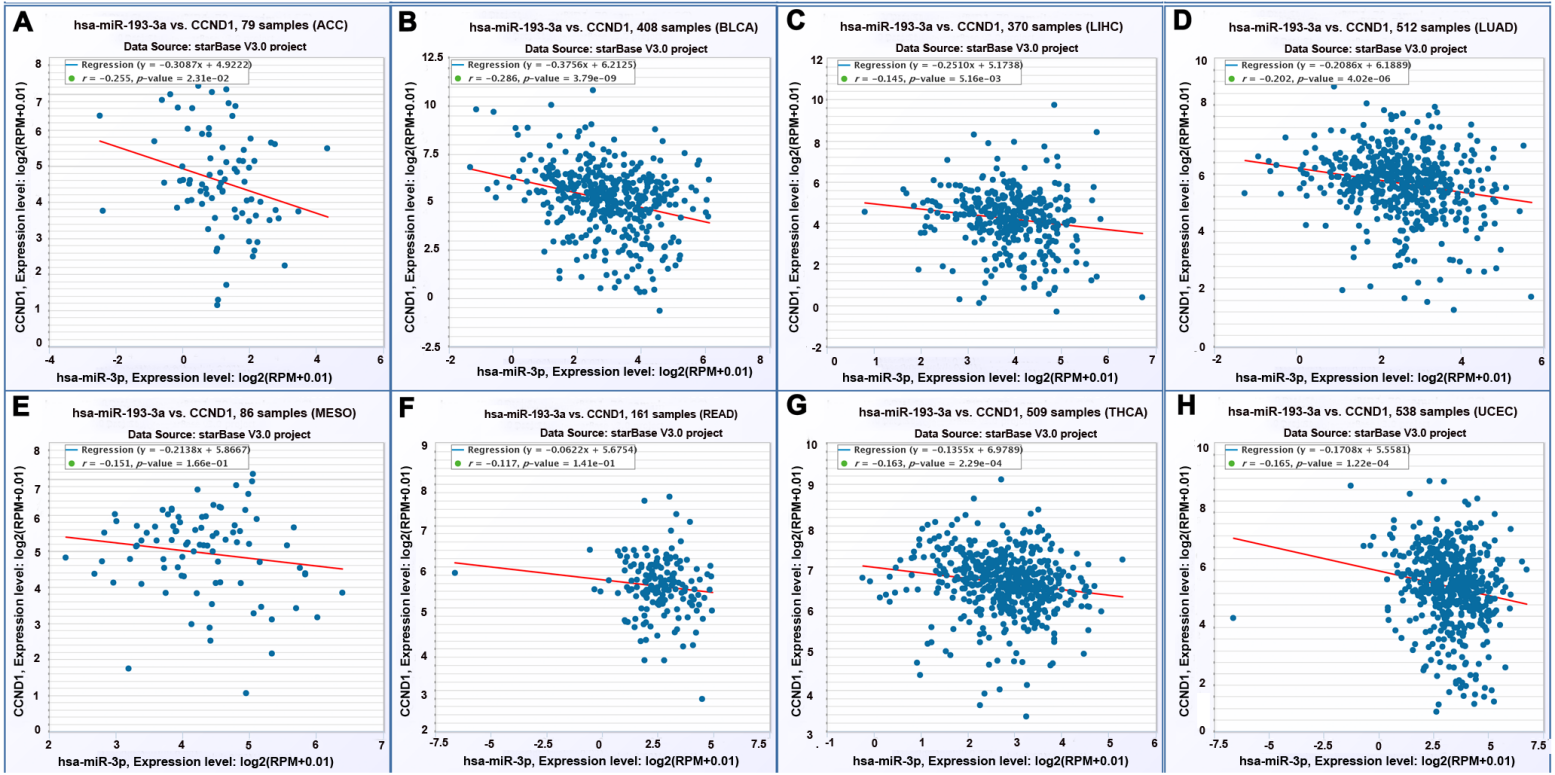
Correlation between miR-193a-3p and CCND1 on the basis of starBase v3.0 pan-cancer analysis project (binding site: chr11: 69467498-69467504[+]). (A) Adrenocortical carcinoma. (B) Bladder urothelial carcinoma. (C) Liver hepatocellular carcinoma. (D) Lung adenocarcinoma. (E) Mesothelioma. (F) Rectum adenocarcinoma. (G) Thyroid carcinoma. (H) Uterine corpus endometrial carcinoma.

The result of the Pearson correlation analysis (two-tailed) showed a trend of negative correlation between miR-193a-3p and CCND1 in the RNA-sequencing database (*P* = 0.002, R = −0.154) (Fig. 6C). To validate this correlation, we acquired the expression of miR-193a-3p and CCND1 in twenty types of cancer from starBase V3.0. There were two binding sites between miR-193-3p and CCND1, namely “chr11: 69467239-69467244[+]” and “chr11: 69467498-69467504[+]”. A negative correlation was observed at both sites in adrenocortical carcinoma, bladder urothelial sarcoma, liver hepatocellular cancer, lung adenocarcinoma, mesothelioma, rectum adenocarcinoma, thyroid tumors, and uterine corpus endometrial cancer (Figs. 7 and 8).

**Table 1:** Significant gene annotations or pathways of GO and KEGG of potential target of miR-193a-3p by DAVID.

**Category**	**ID**	**Term**	**Count**	***P*-Value**
GOTERM_BP_DIRECT	GO:0042147	Retrograde transport, endosome to Golgi	9	1.60E-04
GOTERM_BP_DIRECT	GO:0061025	Membrane fusion	6	3.00E-03
GOTERM_BP_DIRECT	GO:0030968	Rndoplasmic reticulum unfolded protein response	6	3.40E-03
GOTERM_BP_DIRECT	GO:0086002	Cardiac muscle cell action potential involved in contraction	4	5.20E-03
GOTERM_CC_DIRECT	GO:0016020	Membrane	78	1.70E-05
GOTERM_CC_DIRECT	GO:0000139	Golgi membrane	29	1.10E-04
GOTERM_CC_DIRECT	GO:0005829	Cytosol	97	1.60E-03
GOTERM_CC_DIRECT	GO:0005789	Endoplasmic reticulum membrane	32	4.60E-03
GOTERM_MF_DIRECT	GO:0005484	SNAP receptor activity	6	1.70E-03
GOTERM_MF_DIRECT	GO:0017137	Rab GTPase binding	10	3.30E-03
GOTERM_MF_DIRECT	GO:0005515	Protein binding	225	3.70E-03
GOTERM_MF_DIRECT	GO:0042803	Protein homodimerization activity	27	1.50E-02
KEGG_PATHWAY	hsa05214	Glioma	8	9.30E-04
KEGG_PATHWAY	hsa05212	Pancreatic cancer	8	9.30E-04
KEGG_PATHWAY	hsa05205	Proteoglycans in cancer	14	1.10E-03
KEGG_PATHWAY	hsa04012	ErbB signaling pathway	9	1.20E-03
KEGG_PATHWAY	hsa05220	Chronic myeloid leukemia	8	1.70E-03

## Discussion

In this study, we first identified that miR-193a-3p can inhibit the proliferation and promote the apoptosis of HCC through *in vitro* experiments. We then collated and analyzed the expression profile of the HCC sequencing data and found that miR-193a-3p had a low level of expression in HCC. A survival analysis using online databases indicated that HCC patients with higher miR-193a-3p levels tended to have favorable prognoses. We used KEGG, GO pathway enrichment analysis, and PPI network construction to explore the underlying mechanism of the target genes. CCND1 was then defined as the key target gene of miR-193a-3p, which was verified via the dual-luciferase reporter assays. MiRNA-sequencing data for various tumors, including HCC, confirmed that there was a negative correlation between the expression of miR-193a-3p and CCND1. In this paper, it was first discovered that miR-193a-3p played an anti-cancer role in HCC by targeting CCND1, and the relationship between miR-193a-3p and CCND1 expression levels in HCC was explored in depth for the first time.

MiR-193a-3p is a member of the miR-193 family. Recently research has reported the inhibitory effect of miR-193-3p in a variety of tumors. In 2019, Liu et al. (2019) found that a low level of miR-193a-3p expression was related to the increased expression of p21-activated kinase 4 (PAK4), p-Slug, and L1 cell adhesion molecule (L1CAM) in non-small cell lung cancer (NSCLC) and that miR-193a-3p inhibited the metastasis of NSCLC by repressing PAK4, p-Slug, and L1CAM. Through MTT assay and cell colony formation experiments, Yu et al. (2019) showed that miR-193a-3p was downregulated in colorectal cancer cells, while miR-193a-3p’s inhibitors promoted the proliferation and invasion of rectal cells. Recently, many researchers have verified that miR-193a-3p acts as an inhibitor in colon, gastric, and breast cancer because it suppressed the proliferation, migration, and invasion of these cancer cells (Chou et al., 2018; Pekow et al., 2017; Tsai et al., 2016). In addition, miR-193a-3p is involved in the tumorigenicity of nasopharyngeal carcinoma and HCC (Kong et al., 2019; Tsai et al., 2016). In 2015, our team also confirmed the low expression levels of miR-193a-3p in HCC tissues by means of qRT-PCR (Liu et al., 2015). Assuming that miR-193a-3p may exert its biological functions by directly regulating the target genes, we thus predicted the targets using miRwalk 2.0 and constructed a PPI network, in which CCND1 was among the nine most related genes. In this case, we conducted the dual-luciferase reporter assays to verify the direct interaction between miR-193-3p and CCND1. This is the first study to analyze the relationship between the expression levels of miR-193a-3p and CCND1 in HCC. It is ultimately confirmed that miR-193a-3p has an anti-cancer effect in HCC by affecting cell growth and apoptosis *in vitro*.

There are numerous targets of miR-193a-3p, among which serine- and arginine-rich splicing factor 2 (SRSF2), E2F transcription factor 1 (E2F1), and Mcl-1 have been proven to inhibit the development and progression of HCC (Khordadmehr & Shahbazi, 2019; Kwon et al., 2013; Ma et al., 2012; Salvi et al., 2013). In 2012, Ma et al. (2012) found that miR-193a-3p regulated the resistance of HCC to 5-FU via interacting with SRSF2 and E2F1, with SRSF2 being closely related to tumorigenicity of HCC cells and 5-FU resistance. Kong et al. (2019) found that SRSF2 was negatively related to the expression level of miR-193a-3p in nasopharyngeal carcinoma via qRT-PCR. Mcl-1 ectopic expression reversed miR-193a-3p’s promotion of apoptosis, and a reporter assay with a luciferase construct embracing a 3′-untranslated region of Mcl-1 verified that Mcl-1 is a direct target gene of miR-193a-3p (Kwon et al., 2013). All in all, as one of the most effective targets of miR-193a-3p, Mcl-1 is involved in the process of programmed cell death, while miR-193a-3p regulates Mcl-1 and promotes cell apoptosis via inducing the rearrangement of reactive oxygen species and DNA damage (Khordadmehr & Shahbazi, 2019). However, the role of miR-193a-3p with CCND1 in HCC has not been reported.

CCND1, located on chromosome 11q13, encodes the key cell cycle G1 regulation protein Cyclin D1 (Kenny et al., 1999). It is also a proto-oncogene and one of the main regulators of the Wnt signaling pathway (Karimkhanloo et al., 2017). Through combining with cyclin dependent kinase 4 (CDK4) and cyclin dependent kinase 6 (CDK6), CCND1 enables rapid cell proliferation to promote the phosphorylation of retinoblastoma proteins and other substrate (Kenny et al., 1999). In fact, CCND1 plays a role not only in cell cycle but also other carcinogenic effects. Solid tumor models have shown that CCND1 can regulate gene transcription by interacting with specific transcription factors, pigmentation remodeling, and tissue modifying enzymes (Aggarwal et al., 2010; Bienvenu et al., 2010; Fu et al., 2004). CCND1 is overexpressed in epithelial ovarian cancer, colorectal cancer, liver cancer, gastric cancer, nasopharyngeal cancer, and lung cancer, leading to changes in the cell cycle, which, in turn, give rise to the occurrence of tumors (Huang et al., 2014). A study by Tian et al. (2013) demonstrated in 2013 that miR-19b, miR-23b, miR-26a, and miR-92a may promote the proliferation of prostate cancer cells by synergistically regulating the expression of phosphatase and tensin homology, phosphoinositol 3-kinase/Akt, and CCND1 in vitro. There is an article showing that through inhibition of CCND1 expression, miR-193a-3p increases the proportion of G1 prostate cancer cells, thus inhibits the cellular survival and proliferation (Liu et al., 2017). Meanwhile, in breast cancer, the heterotopic expression of miR-193a-3p in cancer cells directly targets mitogen-activated protein kinase 8 (MAPK8), resulting in low CCND1 expression levels (Khordadmehr & Shahbazi, 2019; Uhlmann et al., 2012). Tsai et al. (2016) also reported that the role of miR-193a-3p in inhibiting breast cancer cell migration and invasion was directly related to CCND1. Furthermore, miR-193a-3p can restrain the growth and invasion of gastric cancer cells by targeting CCND1 (Chou et al., 2018). In 2013, Li et al. (2013) found that miR-193a-3p impeded the progression of the myelocyte cycle in acute myeloid leukemia (AML) by targeting CCND1, confirming that high miR-193a-3p expression levels are capable of inhibiting the proliferation of AML cells. Research projects discussing CCND1 in HCC are relatively scarce. Thus, we are the first to study the relationship between miR-193-3p and CCND1 in HCC. At the same time, we also analyzed the miRNA and RNA-sequencing data, determined the base-complementary pairing between miR-193a-3p and CCND1 in miRWalk 2.0, and obtained their correlation in various neoplasms from starBase, confirming that miR-193a-3p and CCND1 were negatively correlated in HCC. Considering the role of miR-193a-3p in inhibiting HCC and the fact that CCND1 inhibits DNA synthesis, cell proliferation, cell colony formation, and hepatoma formation by arresting the G1 phase in the cell cycle (Wu, Lan & Liu, 2019), using miR-193a-3p to inhibit the expression of CCND1 and thus suppress cell proliferation may be a novel strategy for the treatment of HCC. For functional enrichment analyses, the most related pathway was “proteoglycans in cancer”, while the “ErbB signaling pathway” was the second most related pathway. Actually, the ErbB pathway has been shown to participate in the epithelial-mesenchymal transition (EMT) of HCC, and miR-296-5p is able to inhibit EMT in HCC by attenuating ErbB signaling (Shi et al., 2018). In contrast, as a likely target of CCND1 (Karimkhanloo et al., 2017), the Wnt pathway is well-known to be involved in the progression of HCC (Zhu et al., 2019b; Hu et al., 2019; Guan et al., 2019; Zhu et al., 2019a.). However, it was not among the most enriched pathways according to the findings of the KEGG pathway analysis. One explanation for this contradictory phenomenon is that the KEGG pathway was a *in silico* method, which remains to be experimentally verified. Thus, it is interesting to study whether the ErbB signaling pathway is disordered in HCC and whether it surpasses the Wnt pathway in regulating the progression of HCC.

Of course, certain limitations of this research should be noted. Regarding data analysis, because of the insufficient number of chips in the Gene Expression Omnibus (GEO) database, we only analyzed the expression profile of RNA-sequencing data from TCGA. The lack of samples limited the verification of the heterogeneity between clinical parameters and miR-193a-3p or CCND1. Therefore, more samples must be further collated and analyzed to determine the clinical significance of the interaction between CCND1 and miR-193a-3p in HCC. Second, both the qRT-PCR data and the miRNA-sequencing data obtained from TCGA were from tissues; the expression of miR-193a-3p in serum samples has not been determined, which limits its value in diagnosis. Third, because miRNAs have been well-recognized as participating in multiple biological processes by regulating the expression of downstream target genes in a complementary base-pairing manner, the enrichment of the functions and signaling pathways of the target genes did indirectly provide insights into the mechanism of miR-193-3p in HCC. However, the molecular mechanism of the study is relatively lacking. Exactly which signal pathways were involved and how miR-193-3p and CCND1 interacted with these signal pathways remains to be investigated. Finally, regarding *in vitro* experiments, although we carried out in-house qRT-PCR, dual-luciferase reporter assays, and transfection experiments, only one HCC cell line was used. More sophisticated *in vivo* and *in vitro* experiments and more cell lines with multilevel validation are needed for further supplementation.

## Conclusions

Through data mining and in vitro experiments, we determined that miR-193a-3p and CCND1 were negatively correlated in HCC and that miR-193a-3p could inhibit the proliferation and promote the apoptosis of HCC cells by targeting CCND1. This article provided clues for future research concerning the pathogenesis of HCC. We hope this research project can attract more attention to the reliable correlation between CCND1 and miR-193a-3p and thus provide more perspectives on the treatment of HCC.

## Additional Information and Declarations

### Data Availability

The following information was supplied regarding data availability: The raw data is available at Figshare: Fang, Ye-ying (2019): Downregulation of miR-193a-3p is involved in the pathogenesis of hepatocellular carcinoma by targeting CCND1 Peer J #40214. figshare. Dataset. 10.6084/m9.figshare.10646693.v2.
